# Explaining fertility transition of a developing country: an analysis of quantum and tempo effect

**DOI:** 10.1186/2054-7099-1-4

**Published:** 2015-04-21

**Authors:** Ahbab Mohammad Fazle Rabbi, Mohammad Kabir

**Affiliations:** 7 Department of Applied Science, Bangladesh University of Textiles, Dhaka, Bangladesh; 8 Department of Statistics, Jahangirnagar University, Dhaka, Bangladesh

**Keywords:** Fertility transition, Quantum and Tempo effect, Bangladesh

## Abstract

**Background:**

The Total Fertility Rate (TFR) is defined as the average number of births a woman would have if she were to live throughout the reproductive span and bear children at each age at the rates observed in a particular year or period. The current demographic explanation for decline in TFR is primarily attributed to an increase in postponement in pregnancy. Being cross-sectional, fertility measures can be confounded by changes in the timing of births across women’s lifetimes (tempo) and by changes in the numbers of children that they have by the time they end their childbearing (quantum). After a sharp fall in the last two decades, TFR of Bangladesh is now 2.3; whereas the TFR was greater than 3 in the last decade. However, mean age at childbearing showed decreasing trend in the last decade.

**Methods:**

This is a secondary analysis of data from the three consecutive Bangladesh Demographic Health Surveys; BDHS-2004, 2007 and 2011. The method of Bongaarts and Feeney has been applied to estimate the tempo of fertility. Life Table analyses were applied on birth intervals to explain the tempo effect.

**Results:**

There was a sustained decline of the fertility quantum (the number of births per woman) as estimated by the conventional TFR; due to tempo effects during the last three BDHS surveys. Mean age at childbearing also showed decreasing trend in the last decade.

**Conclusions:**

The current study shows the presence of a significant tempo effect with variability of timing in having first or higher order births. If this trend continues, Bangladesh will be able to achieve below replacement level of fertility soon.

## Background

Policy-makers have considered different strategies and interventions aimed at achieving a replacement level of fertility, while researchers search for factors and explanations affecting fertility. The most used indicator of fertility rate is Total Fertility Rate (TFR) which is conceptualized as the average number of children a woman will have over the course of her life if the prevailing age specific fertility rates (period fertility) are assumed to be constant. Demographers and policy makers rely profoundly on the TFR for examining trends in fertility for a number of conveniences [1]. First, the data requirements for the TFR are limited and easy to estimate. Second, the TFR is a synthetic measure of fertility. Third, the TFR is a contemporary measure of fertility and unlike the cohort TFR it can be computed for the year just passed [1]. Using TFR as standard measure of fertility has one important flaw; it is sensitive to shifting in the age pattern or timing of fertility [2]. In conventional use quantum refers to the average number of children born to women in a cohort, and tempo to the timing of births by age of mother within the cohort. Tempo is often measured by the mother‘s mean age at child bearing, but in this paper tempo is measured by the mean ages at child bearing at each birth order [3].

The age pattern of fertility may change for various reasons. Perturbations in the age pattern of childbearing are more subtle, and in recent years are facilitated by effective contraceptive methods. However, a postponement of births not only leads to a rising mean age at child bearing, but it also affects the observed fertility rates and thus the TFR [2, 4, 5]. Specifically, a postponement of fertility leads to a decrease in the observed ASFRs and TFR because events are spread over a longer period of time. Thus, the fertility measures can be confounded by changes in the timing of births across women’s lifetimes (tempo) as well as by changes in the numbers of children that they have by the time they end their childbearing (quantum). This effect was first identified by Ryder [5], but a very simple and effective method to estimate the tempo effect on aggregate level of fertility was invented by Bongaarts and Feeney [6].

Bongaarts and Feeney estimated the effect of timing by decomposing age specific fertility rates applying the parity progression ratio in a calendar year. This method considered the shift in the mean age of childbearing for each birth (parity). Parity-specific analysis provides more detailed insights that cannot be observed using conventional age-specific analysis of fertility. Another important benefit of parity-specific analysis is its capability of providing few threshold measures that can be related more directly to behavioral responses than is the case with age specific fertility rates [6]. Using these measures facilitates deduction about changes that are long-term (quantum) and changes that are temporal (tempo).

The socio-economic and policy changes over the years regarding population and public health sectors in Bangladesh are associated with the fall in fertility. After a fall in the last two decades, TFR is now 2.3 in Bangladesh. If the present trend continues then it is expected that Bangladesh will be able to achieve its demographic goal as envisaged in the population policy. If we divide the country by east and west, BDHS 2011 demonstrates that the west has already achieved replacement fertility but eastern regions such Syllhet and Chittagong are lagging far behind. Health Nutrition Population Sector Development Program (HNPSDP) identified several factors such as utilization of mass media, women empowerment, increased enrolment of girls in education, participation of women in employment and migration. Although significant fertility transition occurred in Bangladesh between 1975 and 2011, Bangladesh has to pass many barriers including high rates of adolescent marriage, lower use of contraception by newly married couples, low education of women and low employment rate of women [7–10]. Declining TFR from 6.3 to 2.3 was not so gradual; Bangladesh has had to pass many barriers. Another possible determinant of this transition may be the emerging success on the fourth goal of MDG; ‘reducing infant mortality rates’; which indirectly contributed to the fall of fertility in the last two decades [11].

Besides decline in the ASFRs, mean age of childbearing also declined since the mid 1970’s which obviously should have some effect on aggregate fertility level of the country. If the TFR changes in a particular year, how we can infer whether fertility rates of the cohorts who are bearing children in that year also changes? It is difficult to predict and interpret changes until relevant cohorts have completed child bearing. A timing change is also known as a tempo change and the tempo effect has a dramatic effect on period fertility rates, but probably has comparatively little effect on cohort rates. Influences on fertility which do affect cohort rates are called quantum effects. However, there have not been enough studies that provide better understanding of recent fertility transition (declining fertility rates from a higher level to replacement level) and its relationship with the change in population size and composition [7]. Few studies have estimated the tempo adjusted TFR of Bangladesh for certain time point [12, 13]. The aim of the present paper is to understand to which extent the fall in TFR can be attributed to the change in age specific fertility rate and to which extent to the period fertility (cross-sectional measure of fertility rate) using three consecutive BDHS data. The decomposed factors of tempo effects like Age-Specific Fertility Rates (ASFR), and mean age at childbearing (MAC) are analyzed to explain tempo adjusted TFR.

## Methods

Data for the analyses in this study come from Bangladesh Demographic and Health Surveys (BDHS). BDHS is a nationally representative survey that was conducted under the authority of the National Institute for Population, Research and Training (NIPORT) of the Ministry of Health and Welfare, Bangladesh and funded by USAID. Three consecutive surveys (2004, 2007 and 2011) have been included in the present study. Stratified Multi-stage Cluster Sampling design was used to collect data and BDHS 2004, BDHS 2007 and BDHS 2011 includes 11400, 10996 and 17749 ever-married women of child bearing age of all six divisions in the country respectively [9].

The Bongaarts and Feeney method has been applied in this paper to estimate the tempo effect [6]. The procedure of Bongaarts and Feeney method depends on construction of TFR which requires the construction of Age-Specific Fertility Rates (ASFRs). DHS generally uses the three years preceding the survey date, thus the numerator is the frequency count of the number of births within 3 years for each of the seven 5-year age groups. The sum of the months each woman spent in each age group over the 36 pre-survey months (women-years of exposure), serves as the denominator. Using this type of coding allows a woman to contribute as little as one month to one age-specific denominator and allows her to divide her exposure to more than one age group (as many as two). The TFR is then computed by adding the ASFRs [9]. Bongaarts and Feeney method used order-specific ASFR and hence TFR to compute the tempo effect. The tempo adjusted TFR (denoted by TFR^*^) for i-th birth order in a particular calendar year y is-



Here MAC_i_ (y-1) denotes the mean age of child bearing of birth order ‘i’ (year 1). If we are estimating tempo effect for the year ‘y’ (year 2); than ‘y-1’ stands for year previous year of year ‘y’ and ‘y + 1’ mean the next year of the year ‘y’ (year 3), i.e. the immediate preceding year to the survey. The mean age of childbearing can be expressed as the weighted average with ASFRs as the weight:



Where, x = 15,20,25,35,40,45; (x + 2.5) represents the midpoint of each age interval and _5_f_x_ represents the age specific fertility rates for five year age group [6]. To compare and contrast highest tempo distorted parity progression, life tables are constructed for corresponding birth intervals using the methods of Rodriguez and Hobcraft [14].

## Results

**Figure 1:**
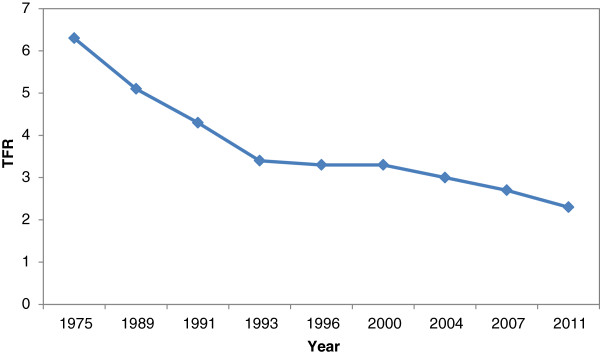
**Fertility Transition of Bangladesh (1975-2011).**

**Figure 2:**
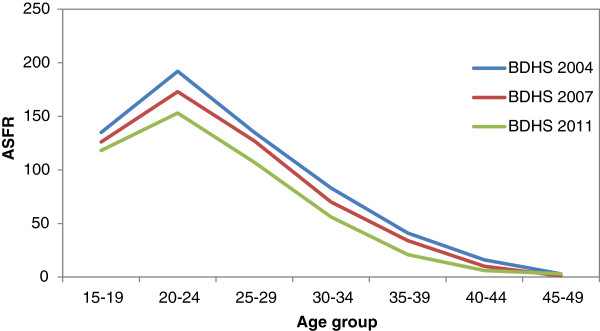
**Trends of ASFR in Bangladesh (BDHS 2004, 2007, 2011).**

TFR transition for the last three decades and ASFR for the last three BDHS is graphically presented below (Figures 1 and 2). Prior to BDHSs, the TFR of the period 1975 and 1989 are taken from Bangladesh Fertility Surveys (BFS); the remaining information is taken from Contraceptive Prevalence Surveys (CPSs).

The decline in TFR over the period of 1975 to 2011 is remarkable for Bangladesh. Rapid decline was observed up to nineties; fertility level plateaued for about a decade before it began to fall again in approximately 2000 BDHS. Adolescent fertility is still high and its contribution is increasing the total specific fertility rate; it is now about one fourth of the total age specific fertility rate which may be explained due to high fertility in the past. ASFR peaked in the age group 20–24 for Bangladeshi women and later age parity progression diminished notably during these three consecutive BDHSs. The details of conventional and tempo adjusted TFR for BDHS 2004, 2007 and 2011 are presented in Table 1.

**Table 1:** **Quantum and Tempo of fertility for Bangladesh (BDHS 2004, 2007, 2011)**

Data	MAC	Birth	No. of	Year 1	Year 2	Year 2	Year 3
		Order	births	MAC _i_	TFR _i_	adj TFR _i_	MAC _i_
**BDHS 2004**		**1**	1291	19.0	0.67	1.42	20.1
		**2**	1066	22.3	0.66	0.89	22.8
	25.5	**3**	770	25.4	0.55	0.59	25.6
		**4**	467	27.5	0.39	0.41	27.6
		**5+**	682	33.5	0.67	0.72	33.6
		**Total**	4276		**2.95**	**4.03**	
**BDHS 2007**		**Birth**	**No. of**	**Year 1**	**Year 2**	**Year 2**	**Year 3**
		**Order**	**births**	**MAC**_**i**_	**TFR**_**i**_	**adj TFR**_**i**_	**MAC**_**i**_
		**1**	1199	19.6	0.81	1.11	20.1
		**2**	979	23.5	0.68	0.75	23.7
	25.2	**3**	617	26.1	0.5	0.92	27.0
		**4**	363	28.6	0.31	0.38	29.0
		**5+**	446	32.6	0.43	0.69	33.4
		**Total**	3604		**2.73**	**3.85**	
**BDHS 2011**		**Birth**	**No. of**	**Year 1**	**Year 2**	**Year 2**	**Year 3**
		**Order**	**births**	**MAC**_**i**_	**TFR**_**i**_	**adj TFR**_**i**_	**MAC**_**i**_
		**1**	1884	19.7	0.71	0.87	20.1
		**2**	1495	23.4	0.65	0.88	23.9
	24.7	**3**	895	26.5	0.49	0.56	26.7
		**4**	441	29.2	0.29	0.33	29.7
		**5+**	423	34.4	0.17	0.18	34.5
		**Total**	5138		**2.31**	**2.82**	

**Figure 3:**
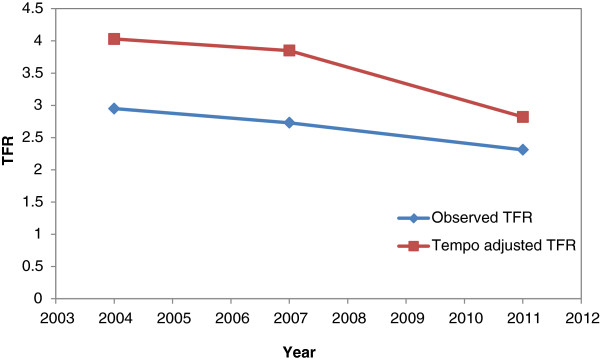
**Observed and Tempo adjusted TFR for Bangladesh (BDHS 2004, 2007, 2011).**

Application of the Bongaarts and Feeney (1998) method suggests the presence of a tempo effect in TFR of Bangladesh, and is significant. The tempo effect for BDHS 2004, 2007 and 2011 were 1.08, 1.12 and 0.51 respectively. From the comparison of the three BDHSs, the highest decline in fertility is observed in the first order birth. For each birth order mean age of child bearing (MAC) by birth order is estimated for the last three BDHSs. In the last three BDHSs (BDHS 2004,2007 and 2011), the MAC for first birth concentrated around 19 to 20 years though the linear trend of increasing MAC_1_ suggest it is more than 19.7 years now indicating a shift in the age specific fertility rates. No change may be observed in first order birth of MAC_1_ in BDHS 2011. For birth order 2, 3 and 4 the MAC have an increasing trend for all three BDHSs. These results indicate the spread of spacing between births for all birth order up to 4th birth order. The conventional and tempo adjusted TFR for BDHS 2004, 2007 and 2011 are presented in Figure 3.

The case of birth order 5 or higher is different from parity progression from birth order 4. The trend of MAC is irregular for higher order births; in BDHS 2007 MAC5+ is lower than MAC5+ for BDHS 2004. This is due to rapid decline in higher order births as Bangladesh approaches a two child family. Tempo adjusted TFR obtained for three combined BDHS (BDHS 1993–94, 1996–97, 1999–2000) was 0.6 [12]; while tempo adjusted TFR for BDHS 2004 is 1.08 in present study [15].In order to find explanations for the obtained pattern of tempo adjusted TFR an analysis of birth intervals was performed and shows an increasing pattern in all three BDHSs. In BDHS 2004 the median birth interval was 39.3 months, which increased to 43.6 months in BDHS 2007 and 47.4 months in BDHS 2011. This clearly suggests the shift in MAC for each order which creates the tempo effect on TFR. But the Figure 2 shows a consistent trend in age specific fertility rates with highest rates in all the three surveys concentrated at age 20–24 indicating mean age child bearing is moving towards younger ages (Figure 2).

Comparing the tempo distortion in different parity progression, it is clear that highest timing effect is observed for first order birth. The median age at marriage was 14 years at BDHS 2004, while it increases to 15 years in next two BDHSs. Age at first birth centered around 17 years in all of three BDHSs (17.3 years, 17.6 years and 17.8 years respectively at BDHS 2004, 2007 and 2011). To examine the marriage to first birth interval, life table technique was applied in all the three BDHSs and the results indicate an increase of cumulative probability of having first birth having longer spacing (Table 2).

**Table 2:** **Life table summary measure for first birth intervals for Bangladesh (BDHS 2004, 2007, 2011)**

Summary measures	BDHS 2004	BDHS 2007	BDHS 2011
**B**_**20**_	0.34	0.37	0.40
**B**_**40**_	0.68	0.71	0.78
**B**_**60**_	0.84	0.85	0.89
**Trimean (T)**	16.25	16.50	17.75
**Spread (S)**	20	21	26
**Median (q**_**2**_ **)**	20	21	22

Here B_20_ means the cumulative probability of having first birth within 20 months of marriage; B_40_ means cumulative probability for 40 months and so on. When the probability is computed for a duration of 60 months (B_60_) it is called ‘quintum’ [14]. The probability of having first birth increased for shorter duration, though the median of first birth interval increased slightly. It should be noted that, most of the births were first order births in all of the BDHSs. Compare to overall birth intervals in BDHSs; first birth interval is comparatively lower. Comparison of three consecutive BDHSs suggests an increase of marriage to first birth interval in Bangladesh. In the BDHS-2004, Tukey’s Trimean was 16.25 for first birth interval which increases to 17.75 months in BDHS-2011. Similarly, spread of births also increased between BDHS-2007 and BDHS-2011 which may be due to the presence of a large tempo effect of first birth.

## Discussion

The aim of the present paper is to understand to which extent the decline in Total Fertility Rate (TFR) can be attributed to the quantum effect and to what extent it is due to tempo effect. The period TFR is one of the most often used indices of fertility, but it is sensitive to variation in the parity distribution of women that results from shifting in the time of childbearing in successive cohorts. The Bongaarts-Feeney tempo adjusted TFR method is one of several attempts to produce better period measure (cross-sectional measures of fertility) of the fertility quantum. Bangladesh, a country of Southeast Asia, has shown significant fertility transition in the last two decades. For Bangladesh, clearly, the impact of timing on the aggregate level of fertility is present. This tempo effect is the result of change in the birth intervals and mean age of childbearing for each birth order. The results indicate the importance of analysis of the timing effect on the aggregate level of fertility which has implications in policy consideration. The primary distortion was caused by an increase in the mean age at child bearing. The analysis shows that tempo adjusted TFR will be half a child higher than the estimates suggest in 2011 BDHS (2.81 as opposed to 2.30). However, the distortion effects of a changing mean age of child bearing by birth order are important in order to understand fertility behavior and its implications on future population growth.

The tempo effect of TFR could be reduced by motivating mothers to widen spacing between consecutive births. Even if Bangladesh achieved replacement fertility by 2015 as envisaged in the population policy of Bangladesh, population growth will continue due to population momentum. The most feasible approaches to reduce the impact of population momentum should be taken into account as policy measures and include delayed age at marriage, delayed age at first birth and wider spacing of successive births.

Despite this, there may be debate about the use of the period measure of TFR. If couples have their children earlier, the mean age at child bearing falls and the period rate will rise. On the other hand, if they have them later, it will fall. Using the Bongaarts and Feeney method one can estimate the effect of shifting in the MAC to the aggregate level fertility only for consecutive years (but not for long periods) [16, 17]. However, the tempo effect of fertility may be distorted due to sampling fluctuations [15]. This limitation is an important policy issue while measuring the tempo effect of TFR might have affected the results of current study.

Life table analysis shows that the highest tempo effect was obtained from age at first parity. The other important consideration from this analysis is the increase in variability in age at first birth and higher order births. Several explanations may be inferred from previous studies, such as lower family planning practices among the adolescents mothers, ‘catching-up’ effects of the educated mothers (having the first birth earlier to overcome the reproductive life span passed before marriage) and so on [10, 18]. This variability of timing in having first or higher order births are thus equally important to understand the fertility transition in Bangladesh.

## Conclusion

The current study shows the presence of a significant tempo effect. Decomposition of parity specific analysis suggests the highest tempo effect on first order birth which is strongly associated with age at marriage. Age at marriage is the most prominent and key-factor explaining fecundability for every sub-group of the study population. Findings from the current study suggest that new policy regarding the present child bearing situation will have important impact on the TFR and maternal and child heath. Further research using cohort measures may identify potentially relevant factors that impact the tempo effect in order to better understand the context.
